# Elevated Preoperative Serum CA19-9 Levels in Patients with Hepatocellular Carcinoma Is Associated with Poor Prognosis after Resection

**DOI:** 10.1155/2013/380797

**Published:** 2013-06-12

**Authors:** Yu-Ling Chen, Chien-Hung Chen, Rey-Heng Hu, Ming-Chih Ho, Yung-Ming Jeng

**Affiliations:** ^1^Graduate Institute of Pathology, National Taiwan University, Taipei 100, Taiwan; ^2^Department of Medicine, National Taiwan University Hospital, Taipei 100, Taiwan; ^3^Department of Surgery, National Taiwan University Hospital, Taipei 100, Taiwan; ^4^Department of Pathology, National Taiwan University Hospital, Taipei 100, Taiwan

## Abstract

Serum levels of the tumor marker CA19-9 have been reported to be elevated in patients with hepatocellular carcinoma (HCC), but its clinicopathologic significance is still unknown. A cohort of 304 patients undergoing surgical resection for HCC and having preoperative CA19-9 data was enrolled in this study. Serum CA19-9 levels were correlated with clinicopathologic factors. Univariate and multivariate analyses were performed to determine the predictors of patient survival. On receiver operating characteristic curve analysis, the cut off value of CA19-9 was determined to be 27 U/mL. One hundred and six patients had preoperative CA19-9 values >27 U/mL. High serum CA19-9 levels did not correlate with patient age, sex, viral status, **α**-fetoprotein level, tumor size, tumor grade, tumor stage, multiplicity, and vascular invasion. Patients with elevated preoperative CA19-9 levels had lower 10-year survival than those without CA19-9 elevation. Multivariate analysis revealed that CA19-9 level, tumor grade, and tumor size are independent prognostic factors for long-term survival. In conclusion, a preoperative CA19-9 value >27 U/mL is associated with poor prognosis after resection for HCC.

## 1. Introduction

Hepatocellular carcinoma (HCC) is the fifth most common malignancy and the third most common cause of cancer deaths globally. It is one of the most common fatal malignancies in Taiwan and many other countries in Asia and Africa [1]. The major risk factors are hepatitis B and hepatitis C infections, cirrhosis of any etiology, and aflatoxin exposure [2]. Despite improved treatment, most patients with HCC die soon after diagnosis because most of them are discovered at late stage, and no effective systemic chemotherapy is available for surgically unresectable tumors. Molecular approaches have revealed the involvement of *p53* and **β*-catenin* mutations in hepatocarcinogenesis [3, 4]. The mutations account for approximately 50% of HCCs. Somatic mutations of other known oncogenes and tumor suppressor genes in HCC are rare. Hence, the molecular mechanisms of HCC remain largely unclear.

CA19-9, also known as sialyl Lewis-a (sLea), is a tumor-associated antigen originally isolated from a human colorectal cancer cell line by Koprowski et al. [5]. It is expressed on the surface of cancer cells as a glycolipid and as an O-linked glycoprotein [6]. CA19-9 is now used as a tumor marker for patients with gastrointestinal cancers, including colorectal, gastric, biliary, and pancreatic cancers [7–10]. Serum level of CA19-9 is elevated in about 75% of cholangiocarcinoma patients [10]. Serum CA19-9 level is also frequently elevated in patients with combined HCC-cholangiocarcinoma, which has two distinct HCC and cholangiocarcinoma components that coexist within a tumor nodule [11]. CA19-9 serum level is also reported to be elevated in a small proportion of HCC patients, and expression of CA19-9 can be detected in tumor cells by immunohistochemistry, but the clinicopathologic significance of its expression is still unknown [12–14].

In this study, we retrospectively analyzed the serum levels of CA19-9 in patients with HCC and studied the clinicopathologic significance and prognostic implication of elevated CA19-9 in HCC patients.

## 2. Patients and Methods

### 2.1. Patients and Samples

From 2000 to 2008, 2182 patients underwent surgical resection for HCC at National Taiwan University Hospital. Of them, 304 patients had preoperative CA19-9 values and were enrolled in this study. The study was conducted according to the regulation of the ethics committee of National Taiwan University Hospital, and the data were analyzed in a blinded manner. After surgery, all patients received laboratory examinations such as serum *α*-fetoprotein (AFP) at 1- to 6-month intervals and ultrasonography of liver at 3–12 month intervals. 94 patients died during the follow-up periods. The median follow-up months of the survivors were 61 months (range: 1 to 132 months).

### 2.2. Histology Study and Tumor Staging

Surgically resected specimens were formalin fixed and paraffin embedded. Histologic sections cut at 5-*μ*m thickness were stained with hematoxylin and eosin and reviewed by one of the authors (Y.-M. Jeng) to determine the tumor grade and stage and exclude mixed hepatocellular and cholangiocarcinoma. The tumor grade was based on the criteria proposed by Edmonson and Steiner [15]. The tumors were staged according to American Joint Committee on Cancer (AJCC) system [16].

### 2.3. CA19-9 Analysis

All serum CA19-9 values were measured using a radioimmunoassay kit manufactured by Abbott Laboratories (Chicago, IL, USA). The recommended upper limit of the normal range for CA19-9 is 37 U/mL. The measurement of CA19-9 was performed within 3 months before operation. If multiple values were available, the maximal values before operation were used for statistical analysis.

### 2.4. Statistical Analysis

Data analyses were carried out using MedCalc statistical software (version 11.4.2.0; MedCalc, Mariakerke, Belgium). A receiver operating characteristic (ROC) curve was constructed to estimate the optimal cut off value of preoperative CA19-9 as the predictor for patient death within three years after operation. Correlation between CA19-9 serum levels and clinicopathologic parameters was evaluated by using the *χ* ^2^ test. Survival rates were calculated by using the Kaplan-Meier method, and difference in survival curves was analyzed by using the log-rank test. The variables with *P* < 0.05 by univariate analysis were subjected to multivariate logistic regression analysis. Multivariate analysis of time to death was analyzed by Cox's proportional hazards models. Two-tailed *P* < 0.05 was considered statistically significant.

## 3. Results

### 3.1. Clinical Features

The patients included 240 man and 64 women with a mean age of 57.7 years (range 24–89 years). Serum hepatitis B surface antigen (HBsAg) was detected in 183 cases and anti-HCV antibody in 85, including 12 positive for both. The overall 1-, 3-, and 5-year survival rates were 86.5%, 74.7%, and 68.5%, respectively. The age, sex, virus status, and stage distribution of the study group are similar to those without CA19-9 values (data not shown). 

### 3.2. Serum CA19-9 Levels in HCC Patients

**Figure 1:**
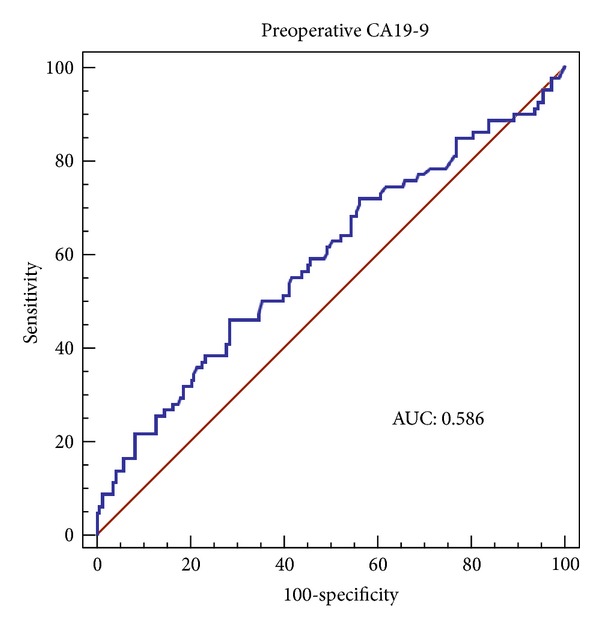
The ROC curve for CA19-9 values and patient death within three years in HCC patients who underwent partial hepatectomy.

Preoperative serum levels of CA19-9 in these patients were determined by radioimmunoassay. An ROC curve demonstrated that a CA19-9 value of 27 U/mL was the optimal cut off point for patient death within three years after operation (Figure 1). The area under the curve (AUC) was 0.586. A total of 106 patients had preoperative CA19-9 values >27 U/mL, among whom 32 (30.2%) patients died within three years. In contrast, only 38 of the 198 patients (19.2%) with CA19-9 values ≤27 U/mL died within three years. In the 106 patients with preoperative CA19-9 values >27 U/mL, 60 patients had CA19-9 values between 27 and 50 U/mL. Twenty-five patients had CA19-9 values between 50 and 100 U/mL. Twenty-one patients had CA19-9 values higher than 100 U/mL. 

### 3.3. Correlation of Serum CA19-9 Level and Clinicopathological Features

**Figure 2:**
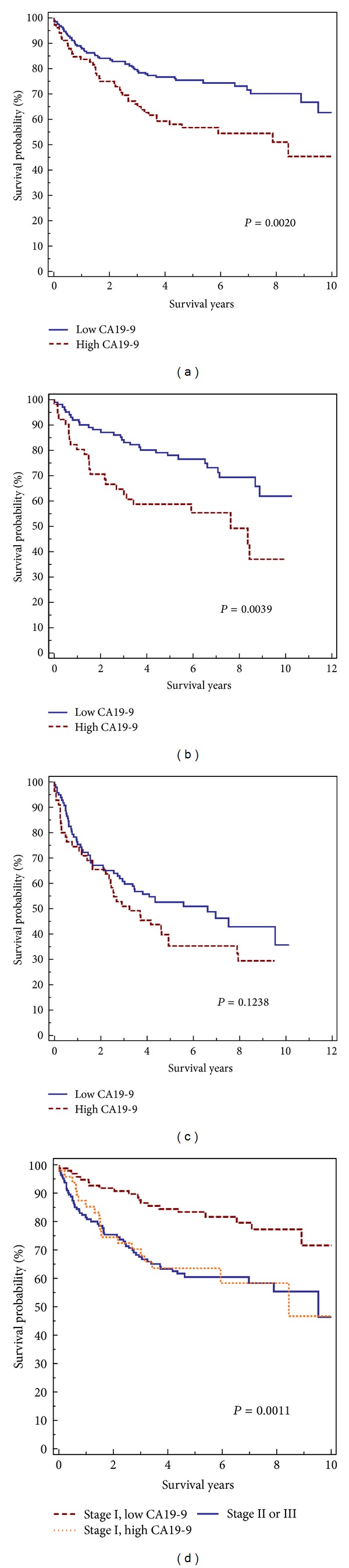
Postoperative survivals calculated by the Kaplan-Meier method. (a) Patients with high CA19-9 serum levels had a significantly lower overall survival than patients with low serum level. (b) Stage I patients with high CA19-9 serum levels had a significantly lower overall survival than patients with low serum level. (c) The survival of patients with high CA19-9 serum levels and stage II and III HCCs was slightly lower than patients with low CA19-9 serum levels, but the difference was not statistically significant. (d) Stage I HCC patients with high CA19-9 levels had similar prognosis with stage II and III patients. Patients with low CA19-9 values and stage I tumors had better prognosis than those with high stage tumor or high CA19-9 values.

We correlated CA19-9 serum levels with a variety of clinicopathological features. As shown in Table 1, elevated preoperative serum CA19-9 level did not correlate with patient age, sex, viral status, *α*-fetoprotein level, tumor size, tumor grade, tumor stage, multiplicity, and vascular invasion. However, patients with elevated preoperative CA19-9 levels had a lower 10-year survival rate than those without CA19-9 elevation (*P* = 0.0020) (Figure 2(a)). The effect of CA19-9 level on patient survival was most obvious in patients with stage I HCC (*P* = 0.0039) (Figure 2(b)). In patients with stage II and III HCCs, those with high CA19-9 levels had slightly lower survival rate than those with low CA19-9 levels, but the difference did not reach statistical significance (*P* = 0.1238) (Figure 2(c)). Most importantly, elevated CA19-9 values in stage I HCC patients indicated similar prognosis with stages II and III patients, whereas patients with low CA19-9 values and stage I tumors had better prognosis than those with high stage tumor or high CA19-9 values (*P* = 0.0011) (Figure 2(d)). 

### 3.4. Identification of Independent Factors for Predicting Long-Term Survival of HCC Patients

Univariate analysis found that *α*-fetoprotein level, CA19-9 level, tumor grade, tumor size, vascular invasion, multiplicity, and tumor stage were significant predictors of long-term overall survival (Table 2). To identify independent prognostic factors, we used these parameters for multivariate analysis. As shown in Table 3, only CA19-9 level, tumor grade, and tumor size emerged as independent prognostic factors.

## 4. Discussion

Many molecular markers were identified to be prognostic factors for HCC [17–19]. Most of these markers are expressed in the tumor tissue, so they can be used only when tissue specimen is available. Few serum markers are available for predicting prognosis of HCC patients. One of the serum markers is *α*-fetoprotein. A high *α*-fetoprotein level correlates with high stage, early recurrence, and poor prognosis of HCC [20]. Serum levels of glypican-3, the absence of vitamin K or antagonist-II (PIVKA-II), manganese superoxide dismutase, and vascular endothelial growth factor have been reported to have prognostic values for HCC patients [21–24]. However, these novel markers are not readily available in most hospitals. 

In this report, we identify CA19-9 as a novel serum marker for predicting survival for patients with HCC. The CA19-9 assay detects a mucin containing a pentasaccharide epitope (fucopentaose II) considered to be a tumor marker in pancreatic adenocarcinoma [25]. Abnormal CA19-9 serum levels were also found in patients with cholangiocarcinoma [26], colorectal cancer [27], gastric cancer [28], and a wide range of benign conditions, such as liver diseases, ascending cholangitis, and pancreatitis [29]. The preoperative CA19-9 level has been reported to be a prognostic factor for pancreatic cancer [30] and cholangiocarcinoma [31]. CA19-9 serum level has been reported to be elevated in HCC patients, but the prognostic implication is still unknown.

In this study, we found that an elevated CA19-9 level is a predictor of shorter long-term survival for HCC patients. The effect of CA19-9 serum levels is most obvious in patients with stage I HCC. Multivariate analysis also identified that CA19-9 is an independent prognostic factor for HCC patients. In contrast with other molecular markers, CA19-9 is a routine cancer marker in daily practice and is available in most hospitals. So it can be applied in clinical practice without difficulty. 

The source of CA19-9 in HCC is still unknown. An elevated CA19-9 serum level is frequently seen in biliary obstruction [32]. CA19-9 is synthesized by normal biliary epithelium. A high CA19-9 level was detected in normal bile [33]. The healthy biliary tract is a secretory pathway for CA19-9. Local compression of the biliary tree by the tumor mass may cause obstruction of small bile ducts and hence produce an increase in the serum level of CA19-9. A subset of hepatocellular carcinoma exhibits partial cholangiocytic differentiation which is characterized by expression of biliary cytokeratin [12]. An elevated CA19-9 serum level strongly associated with this dual-phenotype HCC group [12]. These dual-phenotype HCCs tend to have more aggressive behaviors than pure HCCs [12, 22]. These observations may partially explain the prognostic implication of serum CA19-9 level.

We use 27 U/mL as the cut off point for elevated CA19-9 level in our study, which is lower than the upper limit of the normal range recommended by the manufacturer. Measurement of CA19-9 is usually used in preoperative evaluation and postoperative followup of patients with gastrointestinal adenocarcinomas, which usually produce high levels of CA19-9 by tumor cells. Using a higher cut off can prevent false positive results. In HCC patients, the serum levels of CA19-9 are usually lower than those of gastrointestinal cancer patients. We do not recommend using CA19-9 in screening or followup of HCC patients, but CA19-9 measurement can be used for prognostication in HCC patients, and 27 U/mL may be the optimal cut off for this purpose.

## 5. Conclusion

In conclusion, our study demonstrates that the preoperative serum CA19-9 level is a associated with poor prognosis in HCC patients. This assay is most useful in stratifying stage I HCC patients into different prognostic groups. 

## Figures and Tables

**Table 1:** Univariate analysis of CA19-9 serum level with various clinicopathological features in 304 patients with surgically removed hepatocellular carcinoma.

Variables	CA19-9 serum level (U/mL)		*P* value
	>27	≤27	
Age			0.2560
>55	72	120	
≤55	34	78	
Gender			0.8097
Male	85	155	
Female	21	43	
HBsAg			0.4840
Negative	42	70	
Positive	60	123	
Anti-HCV			0.2552
Negative	68	143	
Positive	34	51	
*α*-fetoprotein (ng/mL)			0.8732
≤200	73	134	
>200	25	50	
Tumor size (cm)			0.6927
<5	61	78	
≥5	45	120	
Grade			0.7288
1-2	40	70	
3~4	65	128	
Vascular invasion			0.9864
No	68	128	
Yes	38	69	
Multiple			0.5695
No	74	145	
Yes	32	52	
Stage			0.7495
I	55	97	
II~III	51	199	

**Table 2:** Univariate analysis of overall survival for clinicopathological parameters.

Clinicopathological features	Hazard ratio (HR)	95% CI for HR		*P* value
		Lower	Upper	
Age (<55 versus ≥55)	0.84	0.54	1.27	0.3943
Gender (Male versus Female)	1.18	0.70	1.94	0.5317
HBV infection ((−) versus (+))	1.14	0.75	1.75	0.5403
HCV infection ((−) versus (+))	0.89	0.57	1.41	0.6468
AFP (<200 ng/mL versus >200 ng/mL)	1.93	1.16	3.20	0.0031
CA19-9 (≤27 U/mL versus >27 U/mL)	1.87	1.21	2.90	0.0020
Grade (1, 2 versus 3, 4)	2.16	1.39	3.34	0.0001
Size (<5 cm versus ≥5 cm)	2.04	1.33	3.13	0.0004
Vascular invasion ((−) versus (+))	1.70	1.09	2.66	0.0094
Multiple ((−) versus (+))	1.79	1.11	2.87	0.0060
Stage (I versus II, III)	1.76	1.17	2.65	0.0057

**Table 3:** Multivariate analysis of overall survival for clinicopathological parameters.

Clinicopathological features	*b*	SE	Exp(*b*)	95% CI of Exp(*b*)	*P* value
AFP (<200 ng/mL versus >200 ng/mL)	0.35	0.24	1.42	0.89–2.27	0.1444
CA19-9 (≤27 U/mL versus >27 U/mL)	0.55	0.22	1.74	1.13–2.68	0.0125
Grade (1, 2 versus 3, 4)	0.49	0.23	1.62	1.04–2.55	0.0342
Size (<5 cm versus ≥5 cm)	0.58	0.23	1.78	1.14–2.79	0.0123
Vascular invasion ((−) versus (+))	0.53	0.40	1.69	0.77–3.69	0.1895
Multiple ((−) versus (+))	0.61	0.34	1.85	0.94–3.61	0.0742
Stage (I versus II, III)	−0.35	0.48	0.71	0.28–1.81	0.4732
